# Supplementary material for: Coffee Polyphenol, Chlorogenic Acid, Suppresses Brain Aging and Its Effects Are Enhanced by Milk Fat Globule Membrane Components
Source: Int J Mol Sci. 2022 May 23;23(10):5832. doi: 10.3390/ijms23105832 (PMC9145055; doi:10.3390/ijms23105832)
Supplement: Supplementary file 1 [file ijms-23-05832-s001.zip › ijms-1724922-supplementary.pdf]

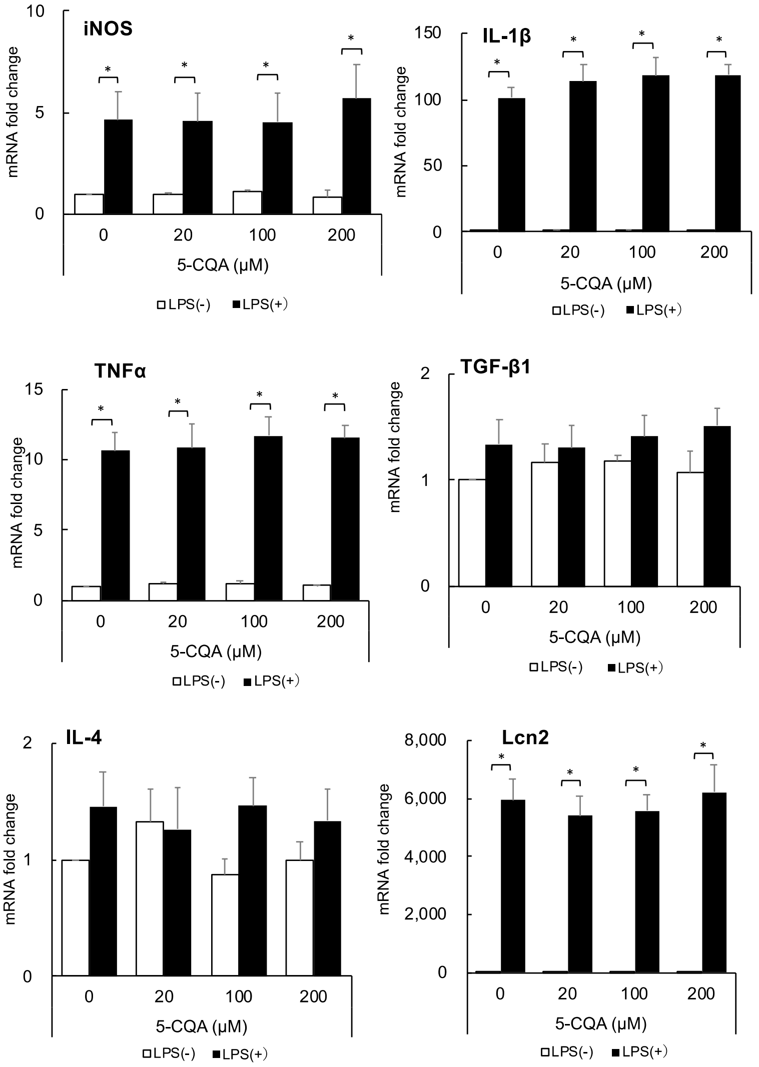

Supplementary Figure S1. Effect of 5-CQA on BV2 cells treated with LPS. Each value represents the mean ± SEM (n = 3–5, *, $p < 0.05$, Fisher's least significant difference test).

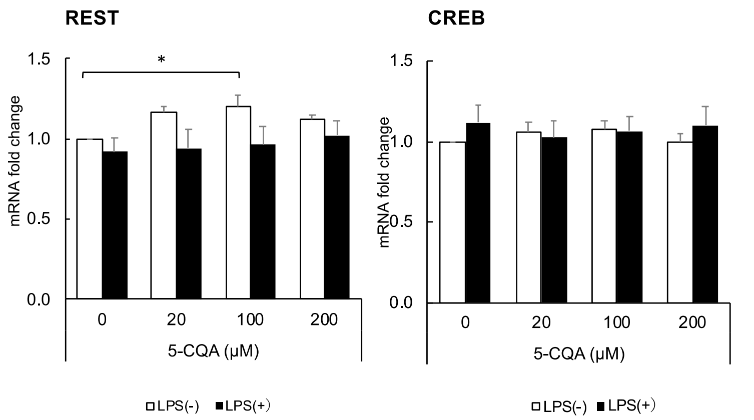

Supplementary Figure S2. Effect of 5-CQA on REST and CREB expression in BV2 cells. Each value represents the mean ± SEM (n = 3–5, *, p < 0.05, Fisher's least significant difference test).

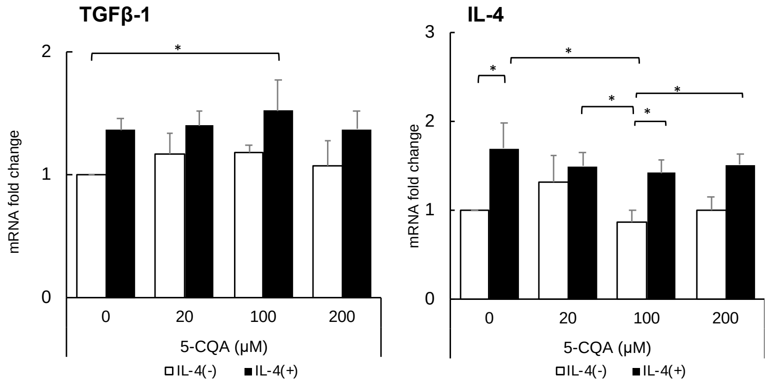

Supplementary Figure S3. Effect of 5-CQA on BV2 cells treated with IL4. Each value represents the mean ± SEM (n = 3–5, *, p < 0.05, Fisher's least significant difference test).
